# Supplementary material for: Spectral clustering of risk score trajectories stratifies sepsis patients by clinical outcome and interventions received
Source: eLife. 2020 Sep 22;9:e58142. doi: 10.7554/eLife.58142 (PMC7508552; doi:10.7554/eLife.58142)
Supplement: Supplementary file 1. [file elife-58142-supp1.docx]

**Title: Spectral Clustering of Risk Score Trajectories Stratifies Sepsis Patients by Clinical Outcome and Interventions Received**

**Supplementary File 1**

**Authors:** Ran Liu, BS^1,2^, Joseph L. Greenstein, PhD^1^, James C. Fackler, MD^3,4^, Melania M. Bembea, MD, MPH, PhD^3,4^, Raimond L. Winslow, PhD^1,2^

**Affiliations:**

^1^Institute for Computational Medicine, The Johns Hopkins University

^2^Department of Biomedical Engineering, The Johns Hopkins University School of Medicine & Whiting School of Engineering

^3^Department of Anesthesiology and Critical Care Medicine, and ^4^Department of Pediatrics, The Johns Hopkins University School of Medicine

**Supplementary Tables:**

**Supplementary File 1A:** Queried Items in eICU

| Feature | Table | ID Column | Labels |
| --- | --- | --- | --- |
| Heart rate | nursecharting | nursingchartcelltypevallabel | ‘Heart Rate’ |
| Systolic BP | nursecharting | nursingchartcelltypevalname | ‘Invasive BP Systolic’ ‘Non-Invasive BP Systolic’ |
| Diastolic BP | nursecharting | nursingchartcelltypevalname | ‘Invasive BP Diastolic’  ‘Non-Invasive BP Diastolic’ |
| Mean BP | nursecharting | nursingchartcelltypevalname | ‘Invasive BP Mean’,  ‘Non-Invasive BP Mean’ |
| Resp. Rate | nursecharting | nursingchartcelltypevallabel | ‘Respiratory Rate’ |
| Temperature | nursecharting | nursingchartcelltypevalname | ‘Temperature (C)’ |
| CVP | nursecharting | nursingchartcelltypevallabel | ‘CVP’, ‘CVP (mmHg)’ |
| PaO_2_ | lab | labname | ‘paO2’ |
| FiO_2_ | respiratorycharting | respchartvaluelabel | ‘FiO2’, ‘FIO2 (%)’ |
| GCS | physicalexam | physicalexampath | ‘GCS’ |
| Bilirubin | lab | labname | ‘direct bilirubin’ |
| Platelets | lab | labname | ‘platelets x 1000’ |
| Creatinine | lab | labname | ‘creatinine’ |
| Lactate | lab | labname | ‘lactate’ |
| BUN | lab | labname | ‘BUN’ |
| Arterial pH | lab | labname | ‘pH’ |
| WBC | lab | labname | ‘WBC x 1000’ |
| PaCO_2_ | lab | labname | ‘paCO2’ |
| Hemoglobin | lab | labname | ‘Hgb’ |
| Hematocrit | lab | labname | ‘Hct’ |
| Potassium | lab | labname | ‘potassium’ |
| Urine | intakeoutput | celllabel | ‘Urine’ |
| Dopamine | infusiondrug | drugname | ‘Dopamine’ |
| Dobutamine | infusiondrug | drugname | ‘Dobutamine’ |
| Epinephrine | infusiondrug | drugname | ‘Epinephrine’ |
| Norepinephrine | infusiondrug | drugname | ‘Norepinephrine’ |
| Ventilator | respiratorycare | n/a | n/a |

**Supplementary File 1B:** Feature Importance in XGBoost

Three metrics of relative feature importance in the XGBoost risk model used to compute risk scores. Gain is the relative contribution of each feature to classification performance. Cover is the relative number of observations in classification trees related to each feature. Frequency is the relative frequency with which each feature occurs in trees in the model.

| **Feature** | **Gain** | **Cover** | **Frequency** |
| --- | --- | --- | --- |
| Lactate | 0.33 | 0.18 | 0.080 |
| SBP | 0.10 | 0.13 | 0.053 |
| Cardio SOFA | 0.057 | 0.081 | 0.016 |
| Creatinine | 0.057 | 0.092 | 0.056 |
| GCS | 0.047 | 0.061 | 0.034 |
| PaO_2_ | 0.037 | 0.018 | 0.046 |
| Urine Output | 0.034 | 0.031 | 0.044 |
| FiO_2_ | 0.031 | 0.050 | 0.043 |
| Resp SOFA | 0.030 | 0.058 | 0.040 |
| Temperature | 0.030 | 0.040 | 0.054 |
| WBC | 0.029 | 0.039 | 0.065 |
| PaCO_2_ | 0.027 | 0.014 | 0.039 |
| Heart Rate | 0.023 | 0.024 | 0.055 |
| Arterial pH | 0.023 | 0.044 | 0.052 |
| MBP | 0.022 | 0.022 | 0.030 |
| Respiratory Rate | 0.017 | 0.016 | 0.041 |

**Supplementary File 1C:** Statistics and demographic information on patients in eICU

We determine clinical state labels using the Sepsis-3 criteria for each patient in the eICU database with at least one entry in the EHR for the items queried in Table S1. We assign each patient to one of three cohorts based on the most severe state reached at any point within their data record. The prevalence of each cohort and the corresponding in-hospital mortality rates are given. We compute statistics on gender, age, length of ICU stay, and Charlson comorbidity index on these cohorts.

| **Most severe clinical state reached** | **No sepsis** | **Sepsis without shock** | **Sepsis leading to septic shock** |
| --- | --- | --- | --- |
| Number of patients | 110,843 | 24,931 | 3,593 |
| Percentage of all patients | 79.5 | 17.9 | 2.6 |
| In-hospital mortality | 4.4% | 7.5% | 31.9% |
| Gender | 52.2% male | 52.4% male | 53.9% male |
| Mean age in years (SD) | 62.4 (17.4) | 66.1 (15.7) | 66.5 (14.9) |
| Median length of ICU stay in days | 1.4 | 2.6 | 4.0 |
| Mean Charlson comorbidity index (SD) | 0.72 (1.16) | 1.12 (1.50) | 1.14 (1.62) |

**Supplementary File 1D:** Charlson Comorbidities in eICU

For the same cohorts as in Table S3, we compute the prevalence of each individual category of comorbidity contributing to the Charlson comorbidity index.

| **Comorbidity** | **Non-sepsis** | **Sepsis** | **Shock** | **Overall** |
| --- | --- | --- | --- | --- |
| Myocardial infarction | 6.4 | 3.7 | 5.7 | 5.9 |
| Congestive heart failure | 8.7 | 15.3 | 13.3 | 10.0 |
| Peripheral vascular disease | 1.4 | 0.7 | 1.7 | 1.3 |
| Cerebrovascular disease | 8.3 | 3.7 | 3.2 | 7.4 |
| Dementia | 0.2 | 0.5 | 0.6 | 0.2 |
| Chronic pulmonary disease | 6.6 | 23.0 | 9.6 | 9.6 |
| Rheumatic disease | 0.3 | 0.4 | 0.2 | 0.3 |
| Peptic ulcer disease | 0.6 | 0.8 | 1.0 | 0.7 |
| Mild liver disease | 2.1 | 3.8 | 9.4 | 2.6 |
| Diabetes without chronic complication | 5.3 | 4.9 | 7.1 | 5.2 |
| Diabetes with chronic complication | 0.0 | 0.0 | 0.0 | 0.0 |
| Hemiplegia or paraplegia | 0.2 | 0.7 | 0.4 | 0.3 |
| Renal disease | 7.5 | 15.1 | 14.3 | 9.0 |
| Any malignancy, including lymphoma and leukemia, except malignant neoplasm of skin | 4.3 | 5.9 | 6.5 | 4.7 |
| Moderate or severe liver disease | 1.0 | 1.1 | 3.5 | 1.1 |
| Metastatic solid tumor | 0.7 | 1.2 | 1.2 | 0.8 |
| AIDS/HIV | 0.1 | 0.2 | 0.4 | 0.1 |

**Supplementary File 1E:** Availability of EHR data in eICU

For each physiological variable queried, we compute the average frequency at which it is reported, as the average length of time between observations, for all observations in patients with at least two measurements of each feature. The proportion of datasets with at least one non-empty entry for each feature is given.

| **Feature** | **Mean hrs/data point** | **Median hrs/data point** | **% datasets with at least 1 data point** |
| --- | --- | --- | --- |
| HR | 2.2 | 1.0 | 92.7 |
| Respiratory Rate | 2.6 | 1.1 | 90.8 |
| Temperature | 4.9 | 4.1 | 96.1 |
| Systolic BP | 2.6 | 1.2 | 92.1 |
| Diastolic BP | 2.6 | 1.2 | 92.1 |
| Mean BP | 2.6 | 1.1 | 88.0 |
| CVP | 3.0 | 1.2 | 13.7 |
| PaO_2_ | 24.3 | 14.7 | 62.1 |
| FiO_2_ | 5.9 | 3.2 | 51.7 |
| GCS | 34.5 | 19.4 | 98.3 |
| Bilirubin | 72.1 | 38.2 | 26.8 |
| Platelets | 24.2 | 22.8 | 97.9 |
| Creatinine | 23.2 | 21.7 | 98.6 |
| Lactate | 28.1 | 11.2 | 63.8 |
| BUN | 23.4 | 21.7 | 98.6 |
| Arterial pH | 24.4 | 14.7 | 61.2 |
| WBC | 24.3 | 22.9 | 98.4 |
| PaCO_2_ | 24.3 | 14.7 | 61.5 |
| Hemoglobin | 23.0 | 22.1 | 98.0 |
| Hematocrit | 23.1 | 22.2 | 98.5 |
| Potassium | 22.5 | 19.8 | 98.1 |
| Urine Output | 9.6 | 4.7 | 79.1 |

**Supplementary File 1F:** Central Tendency Measures of Patient Physiological Data in eICU

For each patient in eICU with at least one entry of the items queried in Table S1, we compute mean and median values for each queried physiological variable. Mean and median are calculated across each non-empty entry in the EHR.

| **Feature** | **Mean Value** | **Median Value** |
| --- | --- | --- |
| HR (bpm) | 89.6 | 88 |
| Respiratory Rate (bpm) | 21.0 | 20 |
| Temperature (C) | 37.5 | 36.9 |
| Systolic BP (mmHg) | 120.1 | 117 |
| Diastolic BP (mmHg) | 63.5 | 62 |
| Mean BP (mmHg) | 78.8 | 77 |
| CVP (mmHg) | 20.1 | 12 |
| PaO_2_ (mmHg) | 106.8 | 86 |
| GCS | 12.9 | 15 |
| Bilirubin (mg/dL) | 1.4 | 0.4 |
| Platelets (k/µL) | 223.1 | 203 |
| Creatinine (mg/dL) | 1.6 | 1.1 |
| Lactate (mmol/L) | 2.7 | 1.8 |
| BUN (mg/dL) | 32.3 | 25 |
| Arterial pH | 7.36 | 7.38 |
| WBC (k/µL) | 12.8 | 11.1 |
| PaCO_2_ (mmHg) | 45.4 | 42 |
| Hemoglobin (g/dL) | 10.0 | 9.7 |
| Hematocrit (%) | 30.7 | 29.8 |
| Potassium (mmol/L) | 4.0 | 3.9 |

**Supplementary File 1G:** Most common causes of infection based on ICD-9 codes specified by Angus et al. (*1*) in eICU

For each patient in eICU with at least one entry of the items queried in Table S1, we determined suspected infection using the ICD-9 codes specified by Angus et al. The prevalence of the categories of infection in Angus et al. most common in the eICU database is given here.

| **Cause of infection – ICD-9 code** | **Prevalence** |
| --- | --- |
| Pneumonia, organism not otherwise specified – 486 | 10.3% |
| Unspecified septicemia – 038.9 | 9.3% |
| Obstructive chronic bronchitis with (acute) exacerbation – 491.21 | 3.1% |
| Urinary tract infection not otherwise specified – 599.0 | 2.2% |
| Cellulitis and abscess of unspecified sites – 682.9 | 1.2% |
| Intestinal infection due to Clostridium difficile – 008.45 | 0.7% |
| Acute pyelonephritis – 590.1 | 0.5% |
| Phlebitis and thrombophlebitis of lower extremities, unspecified – 451.2 | 0.5% |
| Acute and subacute bacterial endocarditis – 421.0 | 0.4% |
| Cutaneous diseases due to other mycobacteria – 031.1 | 0.3% |

**Supplementary File 1H:** Provenance of data (determined from records of ICU stays)

In the *patient* table, under the column *unittype*, the location of each ICU stay is given. We report in this table the frequency of each ICU stay location.

| **ICU Stay Location** | **# in eICU (%)** |
| --- | --- |
| Med-Surg ICU | 113,222 (56.4%) |
| CTICU | 6,158 (3.1%) |
| SICU | 12,181 (6.1%) |
| CCU-CTICU | 15,290 (7.6%) |
| MICU | 17,465 (8.7%) |
| Neuro ICU | 14,451 (7.2%) |
| Cardiac ICU | 12,467 (6.2%) |
| CSICU | 9,625 (4.8%) |

**Supplementary File 1I:** Clusters of risk score following time of early prediction obtained from the MIMIC-III database (Figure S5) stratify by septic shock prevalence and mortality. Differences in median EWT were not statistically significant (Wilcoxon rank-sum test, Bonferroni corrected)

| **Post-Prediction Cluster** | **Size** | **% Septic Shock** | **% Mortality** | **Median Time to Shock Onset (EWT)** |
| --- | --- | --- | --- | --- |
| 1 (High-risk) | 2477 (43.1%) | 82.2% | 44.8% | 5.4 hours |
| 2 | 936 (16.3%) | 43.4% | 24.8% | 7.7 hours |
| 3 (Low-risk) | 2339 (40.7%) | 14.2% | 14.5% | 7.3 hours |
